# Prevalence, Genotype Distribution and Risk Factors for Cervical Human Papillomavirus Infection in the Grand Tunis Region, Tunisia

**DOI:** 10.1371/journal.pone.0157432

**Published:** 2016-06-14

**Authors:** Monia Ardhaoui, Emna Ennaifer, Hajer Letaief, Rejaibi Salsabil, Thalja Lassili, Karim Chahed, Souha Bougatef, Asma Bahrini, Emna El Fehri, Kaouther Ouerhani, Adela Paez Jimenez, Ikram Guizani, Med Samir Boubaker, Nissaf Bouafif ép Ben Alaya

**Affiliations:** 1 Department of Molecular Epidemiology of infectious diseases, Institut Pasteur de Tunis, Tunis, Tunisia; 2 Department of Human and Experimental Pathology, Institut Pasteur de Tunis, Tunis, Tunisia; 3 Department of Epidemiology, Observatoire National des Maladies Nouvelles et Emergentes de Tunis, Tunis, Tunisia; 4 Department of Disease Prevention and Control, European Centre for Disease Prevention and Control (ECDC), Solna, Sweden; Istituto Nazionale Tumori, ITALY

## Abstract

Implementation of Human Papillomavirus (HPV) vaccination should be considered a key cervical cancer prevention strategy in Tunisia, where Pap smear screening is not efficient. This study aims to estimate the prevalence and to identify risk factors associated with HPV infection among women from Grand Tunis, Tunisia. We conducted a cross-sectional study, between December 2012 and May 2013. Eligible women for this study were those aged 18–65 years, sexually active, who sought medical attention at their primary health care centre or clinic in Grand Tunis, Tunisia and who gave written consent. A liquid-based Pap smear sample was obtained from all women using a cervical brush. Only women with betaglobin positive test were further analysed for HPV detection and typing. A nested-PCR of the L1 region was performed followed by reverse line blot hybridization to facilitate the specific detection of 31 HPV genotypes. Multiple logistic regression modeling was used for the analysis of associations between variables with some considered possible confounders after checking for interactions. A total of 391 women were enrolled in this study and 325 out of the 391 cervical samples were positive for the betaglobin test. Overall HPV prevalence was 13.2% [9.8%−17.5%], with the following most prevalent HPV genotypes: HPV6 (40%), HPV40 (14%), HPV16 (12%), HPV52 (9%), HPV31 and HPV59 (7%), followed by HPV68 (4%). Mean age of HPV positive women was 40.7±0.92 years. Independently associated risk factors of HPV infection were smoking (OR:2.8 [0.8–9.6]), low income (OR:9.6 [1.4–63.4), bad housing type (OR:2.5 [1–6.8]), partner with multiple sexual relationship (OR:4.5 [0.9–22.9]) and single women (widowed, divorced, separated, never married) (OR:6.9 [1.1–42.2]). This study provides the first national-based estimate of HPV prevalence in Tunisia. Our findings contribute to the evidence on the current burden of HPV infection, the critical role of sexual behaviour and socioeconomic status and call for increased support for the screening program in Tunisia to prevent cervical cancer. These results allow us to evaluate the cost-effectiveness of vaccine program implementation in Tunisia in future.

## Introduction

Human Papillomavirus (HPV) is the most common cause of sexually transmitted diseases and causes a wide range of pathologies [1, 2]. Although the majority of HPV infections are asymptomatic and self-limiting, the persistent infection with a high-risk HPV (HR-HPV) may cause precancerous lesions that can progress to cancer [1, 3, 4].

In the 1980s, the link between cervical cancer (CC) and HPV was established [5]. During the 1990s, the causal role of HPV was established and accounts worldwide for almost 99% of CC [4–8]. Two vaccines are currently available (Bivalent (HPV16/18 and Quadrivalent HPV6/11/16/18) to protect from HR-HPV-16 and 18 with a good safety and efficacy and a cross-protection against other common HR- HPV types [2, 9–11]. These vaccines have been widely introduced into the national immunization programs in most medium and high- income countries [2, 12].

In Tunisia CC is the third cause of cancer in women resulting in an estimated 1,000 deaths per year often in young women [13]. It represents a major health problem where national screening programs have not shown efficiency [14]. Whereas both vaccines are available in Tunisia, they have not yet been included in the national vaccination program. Such a decision should be informed by estimates of the national HPV prevalence data and a better understanding of the main circulating strains. Three previous Tunisian studies are available reporting different estimates due to differences in the participant recruitment methods, regional variability, and differences in detection tests [15–18]. To our knowledge, no national -based study has previously been conducted in our country.

The present study is a part of a national pilot study. It aims to estimate the prevalence and distribution of HPV genotypes and identify related risk factors among women in the Grand-Tunis region (the capital and main surroundings).

## Materials and Methods

### Study Population

**Fig 1:**
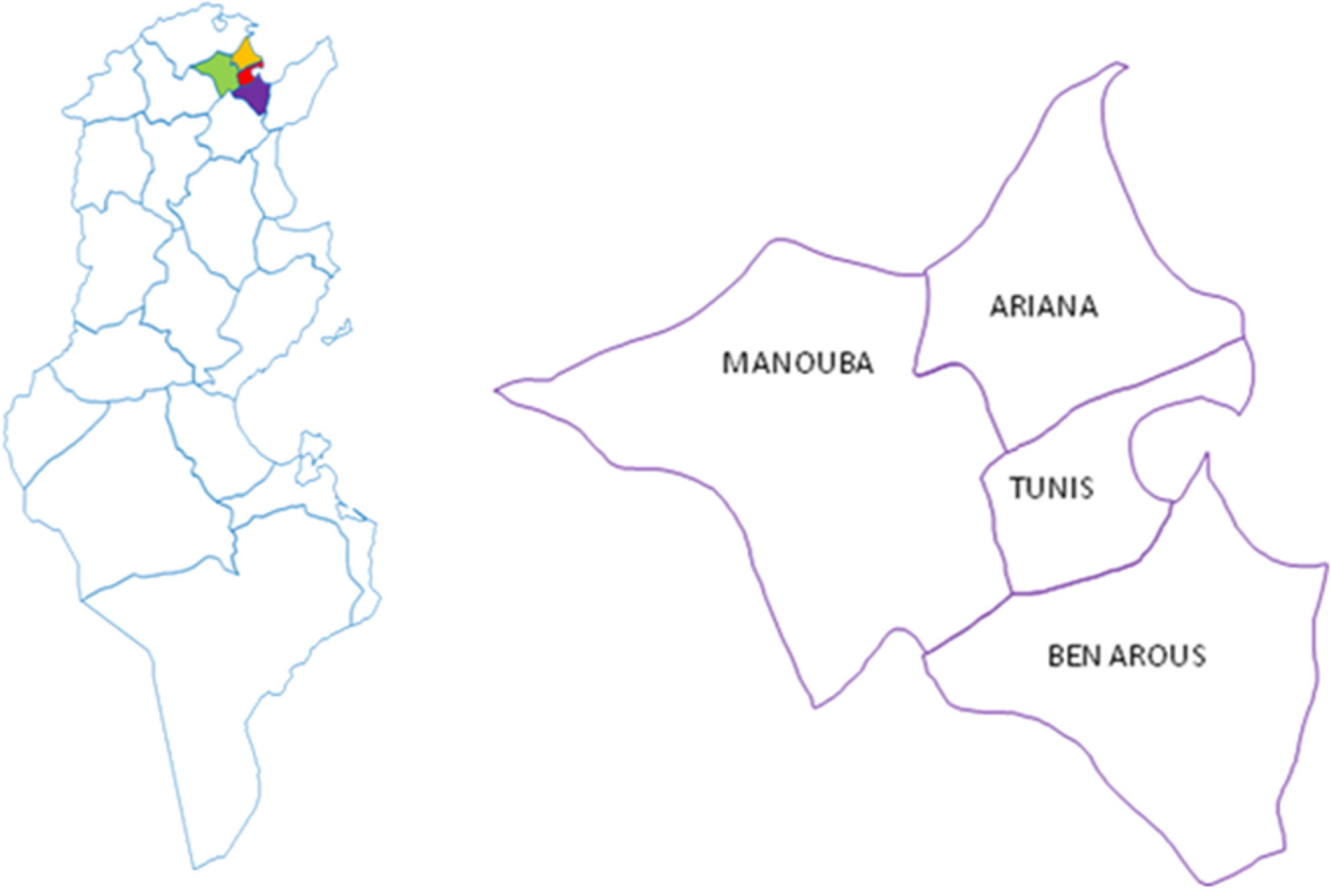
Map showing the 4 governorates of the Grand Tunis region.

A cross-sectional descriptive study was conducted between December 2012 and May 2013. Eligible women were those aged 18 to 65 years old, sexually active resident in the Grand Tunis region, seeking medical attention at their local healthcare centre (CSB) or at a regional reproductive health centre (CRSR) and who gave written consent. The two health centres are considered as a first line healthcare in Tunisia. Selection of CSBs and CRSRs was made proportionally to the size of the governorates between December 2012 and May 2013. As shown in Fig 1, the Grand Tunis region contains four governorates: Tunis, Ariana, Mannouba and Ben Arous.

The sample size (n) was calculated for each governorate (Table 1) using the formula for a simple random sample: (n=DE(Zα22p(1−p)d2)) with a 2% accuracy (d = 2%), an 8% estimated HPV infection prevalence as shown in previous Tunisian studies [17,18], an α error risk of 5% (Zα2=1.96) and a correction factor of dE = 1.5.

**Table 1:** Sample size of each governorate included in the study.

		TUNIS	ARIANA	BEN AROUS	MANOUBA	Total
	**CSB**	78	44	49	34	205
	**CRSR**	75	36	45	30	186
**Total**		153	80	94	64	391

### Data and sample collection

Properly trained medical doctors and midwives conducted face-to-face interviews using a standardized questionnaire, collecting information on socio-demographic status, sexual behaviour of the woman and her partner, reproductive and socio-economic status and medical history. For all women who provided written informed consent, a liquid-based cervical sample was obtained using a cervix brush (Cervexbrush, Easyfix solution Labonord, Templemars, France). Samples were kept at 4°C and sent to the Laboratory of Human and Experimental Pathology at the Pasteur Institute of Tunis (which is the EMRO WHO HPV Reference LabnetLaboratory) in less than one week. Part of the sample was used for a Pap smear and the remaining content stored at -20 C for further use in genomic DNA extraction.

### Cytological Diagnosis

Cytological diagnosis was performed blindly by two pathologists of the laboratory of Human and Experimental Pathology at the Pasteur Institute and the final conclusion was taken by consensus according to the 2001 Bethesda system [19].

### DNA extraction

DNA was extracted from a 200μl aliquot of the suspended cell samples using the QIAamp DNA Blood Mini Kit (Qiagen) according to the manufacturer’s instructions. The quality of extracted DNA was evaluated by betaglobin-specific primers PC04/GH20. Only samples positive for betaglobin were further analysed for HPV detection and genotyping.

### HPV detection

HPV was detected using nested PCR with biotinylated PGMY09/11 primers for the first PCR and GP5+/GP6+ primers for the second PCR. Briefly, 50μl mixture containing 3mM MgCl2, 10 μmol of each primer, 1.5 mM of dNTP (dATP, dCTP, TTP, dGTP), 5μl of the Taq DNA polymerase buffer, 1U of Taq DNA Polymerase, and 10 μl of DNA preparation was aliquoted. The PCR cycling parameters were composed of a 10 minutes initial denaturation at 94°C, followed by 30 amplification cycles of 30 s at 94°C, 1 min at 50°C and 1 min at 72°C, and a final extension step for 7 minutes at 72°C. This reaction was followed by a nested PCR using 10μl of the PGMY PCR product in a reaction mixture containing 50 μmol of GP5+/GP6+ primers, 3mM MgCl2, 1.5 mM each of the dNTP, 1U of Taq DNA Polymerase and 5μl of the Taq DNA polymerase buffer. The PCR cycling parameters comprised of a 10 minutes initial denaturation at 94°C, followed by 40 amplification cycles of 1 minutes at 94°C, 2 minutes at 40°C and 1.5 minute at 72°C, and a final extension step for 7 minutes at 72°C.

### HPV genotyping

Genotyping of positive samples was performed by Reverse Line Hybridization as described in the Human Papillomavirus Laboratory Manual published by the World Health Organization [20]. Briefly, 15μL of denatured PCR products were allowed to hybridize with oligonucleotide probes specific for 31 HPV types (HPV6, 11, 16, 18, 26, 31, 33, 34, 35, 39, 40, 42, 44, 45, 51, 52, 53, 54, 55, 56, 57, 58, 59, 66, 68, 69, 70, 73, 82, 83, and 84) that were immobilized on a Biodyne C membrane using the Miniblotter MN45. The hybridized DNA was detected using Streptavidin peroxydase and Enhanced chemiluminiscent ECL®.

### Statistical analysis

Qualitative variables were described by the simple counts and percentages; quantitative variables by the mean ± SEM. The distribution of HPV genotypes was summarized using frequency distributions. The relation of HPV positivity with demographic, epidemiological and clinical differences was first examined by univariate analysis using the two-sample t test for normally distributed continuous data, the Mann-Whitney test for non-parametric data, the chi-squared test for frequencies and the Fisher’s test and the chi-square test for trend. Odds Ratios (ORs) and 95% confidence intervals (CI) were used to quantify the association between risk factors and positivity for HPV. Multivariable analysis was done using multiple logistic regression to study the relationship between HPV infection (HPV-positive vs. HPV-negative) and the explanatory variables, while adjusting for confounding factors and effect modification if needed. Model building was done using backward procedures. Only variables that retained statistically significant associations with the outcome variables were left in multivariate analyses. A p-value <0.05 was considered statistically significant. Data analysis was performed using SPSS version 22 software.

### Ethical considerations

The study was approved by the Ethics Committee of Institut Pasteur de Tunis and conducted in accordance with Good Clinical Practice, ensuring confidentiality and anonymity. Written informed consent was obtained prior to enrolment of study participants.

## Results

Three hundred ninty-one women were enrolled in this study. Sixty-six out of 391 samples collected were negative for betaglobin and were excluded. HPV detection and typing were performed on the remaining 325 samples. Of note, no significant differences were observed between excluded women and included women in this study (S1 Table).

### Socio-demographic characteristics of study population

Socio-demographic characteristics of the participants are presented in Table 2. The mean age of participants was 40.7± 0.5 years and 54% were over 40 years old. The majority of the participants were married (95%), 17% had no formal education, 64% were unemployed and only 11% had ever smoked. Median age at first sexual intercourse was 23 years (IQR 20–23) and 27% had multiple sexual partners.

**Table 2:** Characteristics of the studied women in the Grand Tunis region, Tunisia.

	N	%
**Age group (years)**		
< = 30	35	10.8%
[30–40[	115	35.5%
[40–50[	127	39.2%
>50	47	14.5%
**Governorate**		
Tunis	127	39.4%
Ariana	70	20.6%
Ben Arous	81	25.5%
Mannouba	47	14.5%
**Marital status**		
Married	308	94.8%
Widowed, divorced, separated, never married	17	5.2%
**Level of education**		
Illiterate	56	17.2%
Primary level	164	50.5%
Secondary and high level	105	32.3%
**Smoking**		
Yes	37	11.4%
No	287	88.6%
**Mean age at sexual intercourse debut (years ±SD)**		24.1±5.7
**Mean number of sexual partners**		1.7±7.0

### HPV prevalence and HPV genotype

HPV prevalence in 325 cases was 13.2% (95% CI, 9.8%-17.5%). Prevalence of HPV infection according to socio-demographic, clinical and behaviour characteristics is summarised in Table 3.

**Table 3:** HPV Prevalence by socio-demographic characteristics, medical history and behaviour.

Demographic Characteristic	Sample Size	HPV Prevalence [95% CI]	P value
**Overall Age**	324	13.2% [9.8%-17.5%]	
**Center**			
CSB	27	62.8%[55.6%-70%]	0.126
CRSR	16	37.2%[29.5%-44.9%]	
**Age group (years)**			
< = 30	35	20.0% [10.4%-33.7%]	0.45
[30–40[	115	12.2% [7.5%-18.6%]	
[40–50[	127	11.0% [6.8%-16.9%]	
>50	47	17.0% [9.2%-28.3%]	
**Governorate**			
Tunis	127	9.4 [5.6%-15.0%]	0.34
Ariana	70	12.9 [7.0%-21.3%]	
Ben Arous	81	17.3 [10.8%-25.9%]	
Mannouba	47	17.0 [9.2%-28.3%]	
**Education level**			
Illiterate	56	12.5 [6.4%-21.9%]	0.17
Primary level	164	16.5 [11.7%-22.4%]	
Secondary and high level	105	8.6 [4.7%-14.5%]	
**Marital status**			
Married	308	11.0% [8.0%-14.7%]	<10^−3^
Widowed, divorced, separated, never married	17	52.9%[32.9%-72.2%]	
**Income Index**			
Below Poverty	74	6.8% [3.0%-13.3%]	0.15
Above Poverty Intermediate Index	136	13.2% [8.7%-19.3%]	
Above Poverty High Index	89	16.9% [10.7%-25.0%]	
**Housing Type**			
Good	97	18.6% [12.2%-26.6%]	0.07
Bad	226	11.1% [8.0%-15.9%]	
**Occupation**			
unemployed	207	11.1% [7.6%-15.7%]	0.11
With regular job	115	17.4% [11.7%-24.6%]	
**Tobacco use**			
Yes	37	29.7%[18.0%-44.1%]	0.02
No	287	11.1%[8.1%-15.0%]	
**Menopause**			
Yes	51	15.7% [8.4%-26.3%]	0.6
No	274	12.8% [9.4%-16.9%]	
**Pregnancy**			
Yes	9	33.3% [13.7%-66.0%]	0.06
No	296	12.2% [9.0%-16.1%]	
**Contraception**			
Yes	88	14.8% [9.0%-22.6%]	0.6
No	231	12.4% [8.9%-17.0%]	
**Medical history of chronic disease**			
Yes	102	10.8%[6.3%-17.3%]	0.4
No	223	14.3%[10.4%-19.2%]	
**Surgical history**			
Yes	126	15.1%[10.0–21.6%]	0.4
No	198	12.1%[8.3%-16.9%]	
**casual sexual relation the last 12 months**			
Yes	12	50.0%[27.7%-72.3%]	<10^−3^
No	288	11.5%[8.3%-15.3%]	
**Sexually transmitted infection history**			
Yes	87	16.1%[10.0%-24.2%]	0.35
No	238	12.2%[8.7%-16.6%]	
**Multiple sexual intercourse of partner**			
Yes	29	31.0% [17.9%-47.3%]	0.001
No	277	10.5% [9.1%-17.3%]	
**age at first sexual intercourse**			
≤ 18 years	39	20.5% [17.8%-24.0%]	0.13
>18 years	286	12.2% [11.2%-13.5%]	
**Multiple sexual partners**			
Yes	18	33.3% [29.1%-38.4%]	0.015
No	236	11.0% [10.0%-12.3%]	

**Fig 2:**
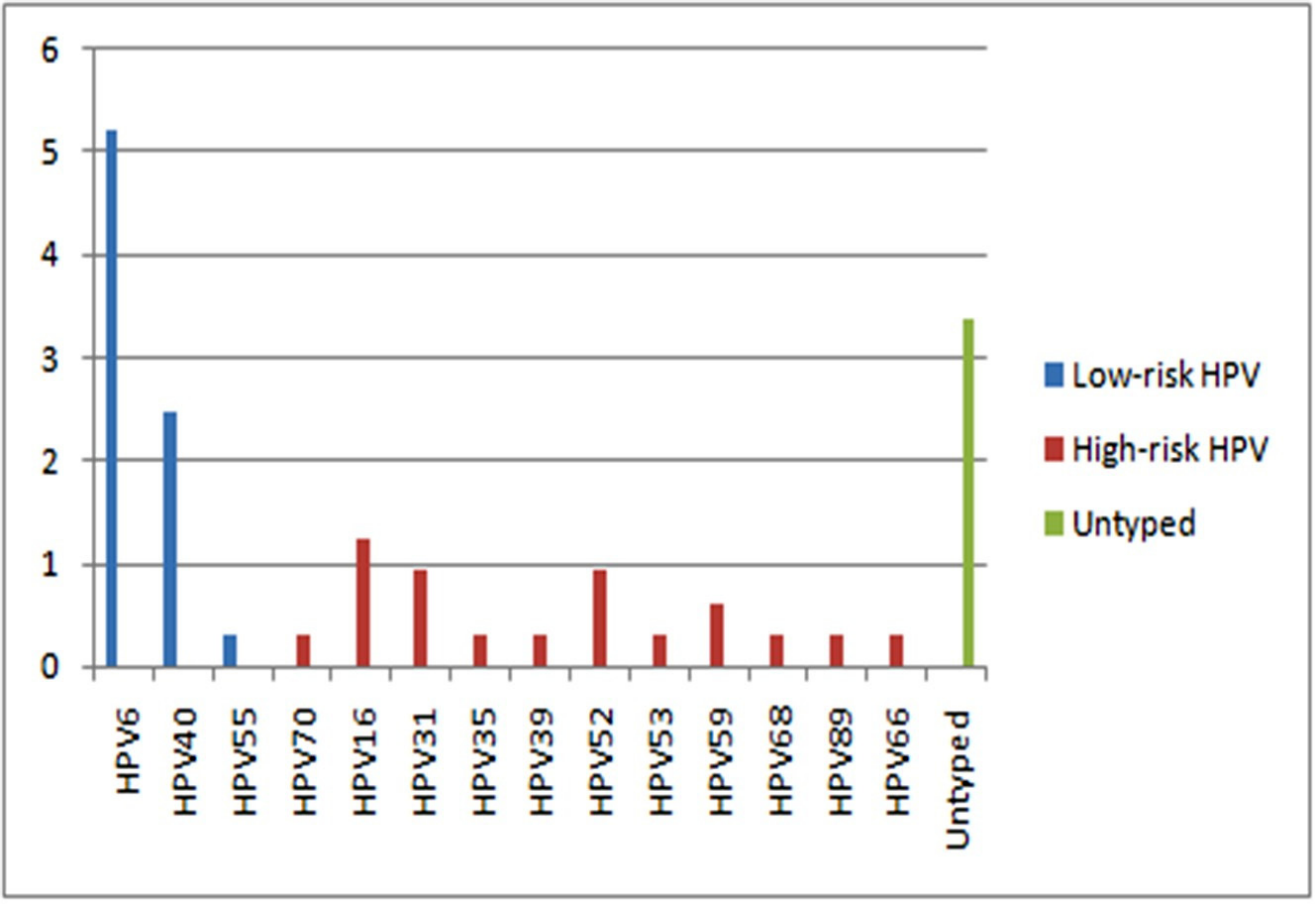
HPV Type prevalence in Grand Tunis Region.

The prevalence of HR-HPV and low-risk HPV (LR-HPV) types were 3.1% (95% CI, 1.5%-5.2%) and 6.5% (95% CI, 4.0%-9.5%), respectively. Multiple infections were detected in only 18% of positive samples (8/43) and single infections in 55.8% (24/43). Of the 8 multiple HPV infected samples, 5 were infected with 2 HPV types, 2 were infected with 3 HPV types, and 1 was infected with 4 HPV types. Multiple infections with both HR-HPV and LR-HPV types were the most common (62.5%). mixed infections with HR-HPVs were observed in 25% of cases while mixed infections with LR-HPVs were seen in 12.5% of cases. The most common HR-HPVs were HPV16 (1.2%; 95% CI, 1.0%-1.6%), HPV31 and HPV52 (0.9%; 95% CI, 0.8%-1.2%). HPV6 (5.2%; 95% CI, 4.6%-6.0%) and HPV40 (2.5%; 95% CI, 2.1%-3.0%) were the most common in LR-HPV. The prevalence of detected HPV types is shown in Fig 2.

#### HPV distribution according to age

**Fig 3:**
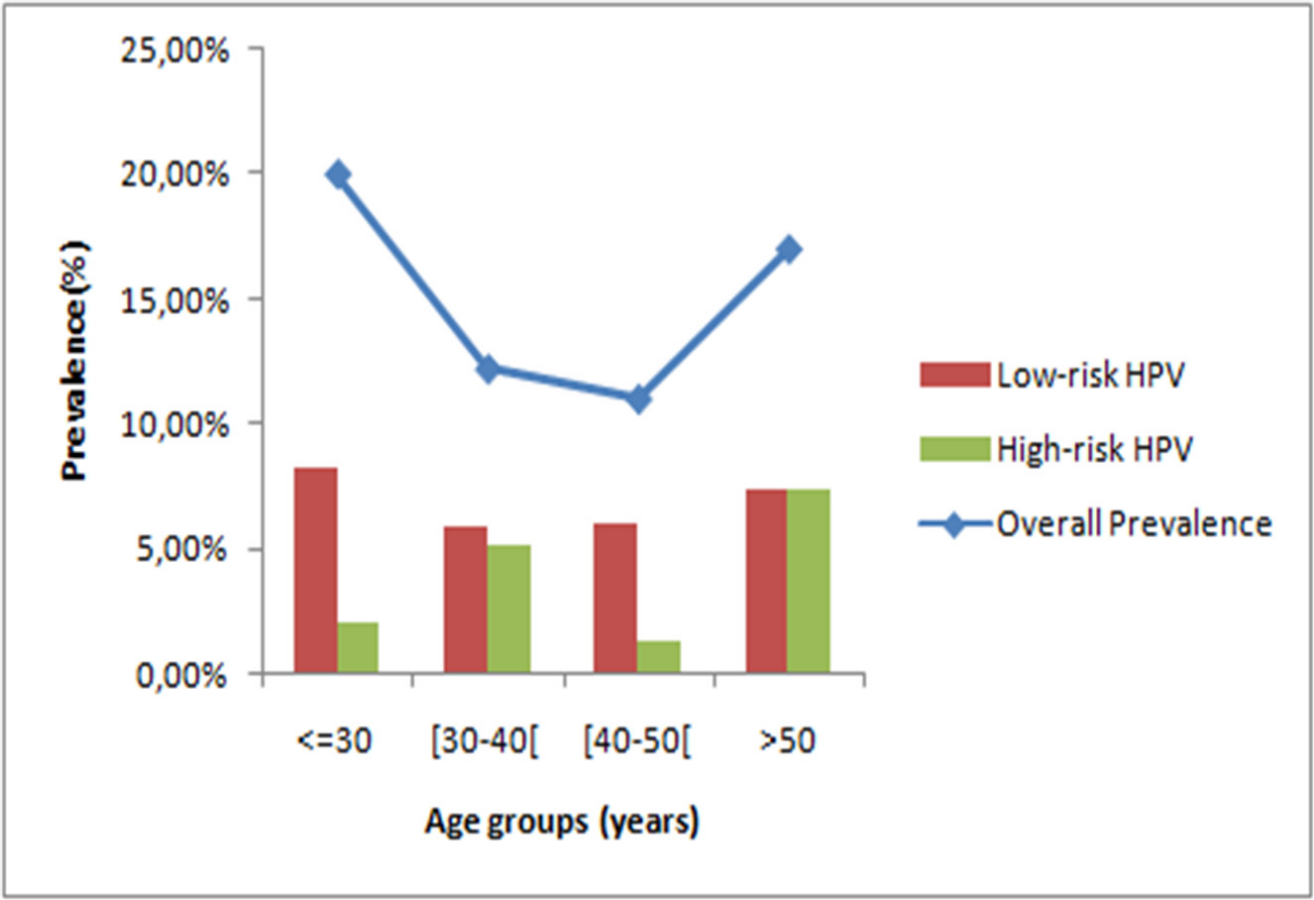
HPV prevalence according to age groups.

Prevalence of HPV infection was highest among women aged less than 30 years (20%; 95% CI, 10.4%-33.7%), followed by a non-significant decline in HPV prevalence in women aged 30 to 50 years and a new increase after 50 years of age (p = 0.45). There was a statistically significant difference between low- and high-risk HPV prevalence trends by age (Fig 3). Prevalence of HR-HPV types increased significantly with age (p = 0.04), while this was not the case with LR-HPV prevalence (p = 0.8) as showed in Fig 3.

The distribution of detected genotypes according to age groups showed that HPV 16 was the most common type in women aged 31–40 and over 50 years-old. HPV31 was present in all age groups except in women aged 41–50 year. Here HPV6, HPV40, and HPV35 were the major genotypes (Table 4).

**Table 4:** HPV type distribution according to age groups.

	Age groups			
HPV Genotypes	< = 30	31–40	41–50	>50
**HPV6**	11,8%	41,2%	35,3%	11,8%
**HPV40**	37,5%	25,0%	25,0%	12,5%
**HPV55**	0,0%	0,0%	0,0%	100,0%
**HPV70**	0,0%	0,0%	100,0%	0,0%
**HPV16**	0,0%	50,0%	0,0%	50,0%
**HPV31**	33,3%	33,3%	0,0%	33,3%
**HPV35**	0,0%	0,0%	100,0%	0,0%
**HPV39**	0,0%	100,0%	0,0%	0,0%
**HPV52**	33,3%	33,3%	0,0%	33,3%
**HPV53**	0,0%	100,0%	0,0%	0,0%
**HPV59**	0,0%	50,0%	0,0%	50,0%
**HPV68**	0,0%	0,0%	100,0%	0,0%
**HPV89**	0,0%	0,0%	100,0%	0,0%
**HPV66**	0,0%	0,0%	100,0%	0,0%

#### HPV distribution according to cytology

A total of 27 (8.3%) cervical cytology samples were inadequate. For those with adequate results, Pap smear was normal for 115 women (35.4%, 95% CI, 33.4%-44.4%). It was atrophic or inflammatory for 141 women (43.4%, 95% CI, 41.9%-52.8%), Low-squamous intraepithelial lesions (LSIL) were seen for 38 women (11.7%, 95% CI, 9.5%-16.7%) and High-squamous intraepithelial lesion (HSIL) and Atypical glandular undetermined significance (AGUS) accounted for two cases (0.6%, 95% CI, 0.2%-1.9%). HPV prevalence was 16.5% (95% CI, 11.0%-23.6%) in normal smear, 10.6% (95% CI, 6.6%-16.1%) in atrophic or inflammatory smears and 18.4% (95% CI, 9.6%-31.3%) in LSIL. HPV infection in HSIL accounted for 50% of cases and all were single HPV31 infections. The 2 cases of AGUS were HPV negative.

### Risk factors for HPV infection

Associated factors with HPV infection in univariate analysis were marital status, tobacco use, sexual behaviour and partner sexual behaviour (Table 5).

**Table 5:** Univariate analysis of associated factors with HPV infection.

Characteristic	OR [95% CI]	P value*
***Marital status***		
Married	Ref	
Widowed, divorced, separated, never married	6.9 [1.1–42.2]	<10^−3^
***Income Index***		
Below Poverty Index	Ref	
Above Poverty Intermediate Index	9.6 [1.4–63.4]	0.019
Above Poverty high Index	22.1 [2.8–175.5]	0.003
***Housing Type***		
Good	Ref	
Bad	2.5 [1–6.8]	0.05
***Smoking***		
Yes	2.8 [0.8–9.6]	0.003
No	Ref	
***Partner with multiple sexual encounters outside relationship***		
Yes	4.5 [0.9–22.9]	0.07
No		
***Age at sexual intercourse debut***		
≤ 18 years	2.7 [0.8–9.5]	0.12
>18 years	Ref	

* χ2 Wald

Multivariate analysis suggests that marital status, partner with multiple sexual encounters outside the stable relationship, housing type and income index were independently associated with HPV infection (Table 6). However, the age at sexual intercourse debut and the increasing number of sexual encounters of the partner were not significant in the multivariate model.

**Table 6:** Multivariable analysis of associated factors with HPV infection (N = 219).

Characteristic	AOR [95% CI]	P value*
***Marital status***		
Married	Ref	
Widowed, divorced, separated, never married	6.9 [1.1–42.2]	<10^−3^
***Income Index***		
Below Poverty Index	Ref	
Above Poverty Intermediate Index	9.6 [1.4–63.4]	0.019
Above Poverty high Index	22.1 [2.8–175.5]	0.003
***Housing Type***		
Good	Ref	
Bad	2.5 [1–6.8]	0.05
***Smoking***		
Yes	2.8 [0.8–9.6]	0.003
No	Ref	
***Partner with multiple sexual encounters outside relationship***		
Yes	4.5 [0.9–22.9]	0.07
No		
***Age at sexual intercourse debut***		
≤ 18 years	2.7 [0.8–9.5]	0.12
>18 years	Ref	

* χ2 Wald

## Discussion

Baseline information on HPV prevalence and genotype distribution is crucial to evaluate the impact of HPV vaccines and inform the best approach for cervical cancer prevention. Our cross-sectional study estimated HPV prevalence in women residing in the Grand Tunis region and is a part of the first large-scale national-based epidemiological study on the prevalence of HPV infection and genotype distribution.

In this study, the prevalence of HPV infection in the Grand Tunis region was estimated as 13.2%. The few previous studies in Tunisia that focused on a small series of participants in cervical screening programs have shown divergent results, that is 43.8% among Tunisian female prostitutes [16], 7.8% in the reproductive health centre of Ariana [17] and 6.5% in Urban Tunis region [18]. Heterogeneity with regards to the methods for participant selection and the representativeness in the population and the lack of epidemiological and behavioural data make any comparison with the current study findings difficult. Several meta-analyses [21–23] confirmed that HPV infection varied by geographical area, age, life style and socio-economic status. Difference in HPV prevalence might also partly be attributed to the difference in sample population and methods used. The HPV prevalence estimated in our study is similar to that reported in a study in Morocco [24], but higher than that reported in other North African countries, namely 6.3% in Algeria [25] and 10.3% in Egypt [26].

In this study, HR-HPV accounted for about half of all HPV infections, with the most prevalent HR-HPV being HPV-16, 31, 52 and 59. Comparing with reported series, HPV16 was the most common genotype [24, 25,26] and HPV52 and 31 were the second most common genotypes in Africa and Europe, respectively [21–23]. Of note, HPV18 the second most frequently detected HR-HPV worldwide [21–23] was surprisingly scarce in our study population. In our study, almost four per cent of the samples could not be genotyped. This may be due to the fact that some samples had low viral load and/or some HPV types were not included in the tested probes [27].

Two peaks of infection by age appear clearly in our results. One peak in ages less than 30 years, which could represent the beginning of sexual activity, and another peak in ages greater than 50 years. This second peak may partly be explained by a relative lack of viral clearance and insufficient adaptive immune responses at this age caused by hormonal changes, contributing to HPV persistence or reactivation of latent HPV infections [28–31]. Our results were consistent with the meta-analysis conducted by Bruni et al. [22] wherein the age distribution of cervical HPV infection showed a bimodal curve with a first peak at younger ages (<25 years), that is just after sexual intercourse debut, a low prevalence plateau within the middle age groups (30–40 years), and a rebound at older ages (>45 years). Previous studies showed that the prevalence of multiple HPV infection was the highest in the oldest age group [32–33]. A possible explanation is that the immune response to HPV infection in older women is relatively low, and immunomodulation of the virus is suppressed [30–34]. These results indicate that women in the young and old age group need more clinical surveillance and management of HPV infection.

In this study HPV infection in normal Pap smears accounted for 16.5% (95% CI, 11.0%-23.6%). This prevalence is similar to reported series in Africa (7.3%-37.1%), America (4.6%-42.2%) and Europe (8.5%-22.7%) [22].

In our study, risk factors were shown to be specific: the better the income, the greater the risk of HPV infection. Several risk factors related to HPV infection and persistence, such as age, smoking, age at first sexual relation and multiple sexual partners, are reported in the world with differences due to the specificities of each studied population [1, 23, 25, 29]. In contradiction with our data, low-income level increases the risk of HPV infection, probably related to lack of access to proper care, which facilitates infection and persistence of HPV and an increasing risk of turning into cancer [35–37].

It is established that smoking increases the risk of HPV infection and persistence through a vulnerability of immune system which facilitates infection and persistence of HPV and an increasing risk of turning into cancer [38]. In our study, current smokers seem to be almost three times more at risk of getting HPV infection compared to non-smokers. However, the association is not significant, probably due to lack of power of a limited sample size. In the literature, it has been shown that smoking was associated with an increased prevalence of HPV [29, 38–40].

Various aspects of sexual behaviour were reported to be related to the acquisition of HPV infection [23, 29, 40]. In the present study, the marital status appears as a factor associated with HPV infection. Single or divorced women are more often infected than married ones (52.9% versus 11%). This could be explained by a different way of life that could lead them to have more than one sexual partner [40]. However, unlike previous studies [40–42], neither age at the first intercourse nor the number of sexual partner, were significantly associated with HPV infection. These findings need to be considered in light of the extremely sensitive nature of this question and possible inaccurate answer, leading to exposure misclassification bias.

## Conclusion

HPV infection prevalence in the Grand Tunis region was 13.2% and is larger than previous estimates in Tunisia. HR-HPV types accounted for half of all infections and were mainly HPV-16, 31, 52 and 59. Smoking, sexual behaviour of partner and high level income were the main risk factors. This study is the pilot phase of a national survey which aims to assess the national HPV prevalence in the whole of Tunisia and provide baseline data to enhance the understanding of HPV infection. Those findings will inform recommendations to optimize HPV screening efforts for high-risk genotypes, the most affected age groups and to evaluate the cost-effectiveness of vaccine program implementation in Tunisia in future.

## Supporting Information

S1 Table Comparaison between betaglobin positive and betaglobin negative samples. (DOC)

## References

[1] Sudenga S, Shrestha S. Key considerations and current perspectives of epidemiological studies on human papillomavirus persistence, the intermediate phenotype to cervical cancer. International Journal of Infectious Diseases. 2013; 17(4):e216–e220. 10.1016/j.ijid.2012.12.027 23453716 PMC3602330

[2] WHO. Human papillomavirus vaccines: WHO position paper. Wold Health Organisation 2014.

[3] Doorbar J, Quint W, Banks L, Bravo I, Stoler M, Broker T, et al The Biology and Life-Cycle of Human Papillomaviruses. Vaccine. 2012; 30: F55–F70. 10.1016/j.vaccine.2012.06.083 23199966

[4] Zur Hausen H. Human genital cancer: synergism between two virus infections or synergism between a virus infection and initiating events?. The Lancet. 1982; 320(8312):1370–1372. 10.1016/s0140-6736(82)91273-9 6129466

[5] Walboomers J, Jacobs M, Manos M, Bosch F, Kummer J, Shah K, et al Human papillomavirus is a necessary cause of invasive cervical cancer worldwide. J Pathol. 1999;189(1):12–19. 10451482 10.1002/(SICI)1096-9896(199909)189:1<12::AID-PATH431>3.0.CO;2-F

[6] Dillner J. Prevention of Human Papillomavirus–Associated Cancers. Seminars in Oncology. 2015;42(2):272–283. 10.1053/j.seminoncol.2014.12.028 25843731

[7] IARC working group. Human papillomavirus. IARC Monographson the evaluation of carcinogenics risk to humans Vol.64 Lyon: International agency for research on cancer; 1995.

[8] Bosch F, Lorincz A, Munoz N, Meijer C, Shah K. The causal relation between human papillomavirus and cervical cancer. Journal of Clinical Pathology. 2002;55(4):244–265. 11919208 10.1136/jcp.55.4.244 PMC1769629

[9] Einstein M, Baron M, Levin M, Chatterjee A, Edwards R, Zepp F, et al Comparison of the immunogenicity and safety of Cervarix ™ and Gardasil ® human papillomavirus (HPV) cervical cancer vaccines in healthy women aged 18–45 years. Human Vaccines. 2009;5(10):705–719. 19684472 10.4161/hv.5.10.9518

[10] Villa L. Prophylactic HPV vaccines: Reducing the burden of HPV-related diseases. Vaccine. 2006;24:S23–S28. 16194583 10.1016/j.vaccine.2005.09.001

[11] De Vincenzo R, Ricci C, Conte C, Scambia G. HPV vaccine cross-protection: Highlights on additional clinical benefit. Gynecologic Oncology. 2013;130(3):642–651. 10.1016/j.ygyno.2013.05.033 23747835

[12] Jumaan A, Ghanem S, Taher J, Braikat M, Awaidy S, Dbaibo G. Prospects and Challenges in the Introduction of Human Papillomavirus Vaccines in the Extended Middle East and North Africa Region. Vaccine. 2013;31:G58–G64. 10.1016/j.vaccine.2012.06.097 24331821

[13] Ferlay J, Shin H, Bray F, Forman D, Mathers C, Parkin D. Estimates of worldwide burden of cancer in 2008: GLOBOCAN 2008. International Journal of Cancer. 2010;127(12):2893–2917. 10.1002/ijc.25516 21351269

[14] Dimassi K, Hleili W, Saidi O, Ben Alaya N, Ben Romdhane H. Knowledge and uptake of genital cancer screening methods among Tunisian women. International Journal of Gynecology & Obstetrics. 2015;128(3):268–269. 25468051 10.1016/j.ijgo.2014.09.019

[15] De Marco F, Houissa-Kchouk F, Khelifa R, Marcante M. High-risk HPV types in Tunisia. A pilot study reveals an unexpectedly high prevalence of types 58 and 82 and lack of HPV 18 among female prostitutes. J Med Virol. 2006;78(7):950–953. 16721861 10.1002/jmv.20646

[16] Ennaifer E, Tounsi H, Ben Aissa R, Kalai K, Fehri E, Laassili T. Screening of cervical HPV infection at the reproductive health centre of ariana. Tunis Med. 2014 4;92(4):253–7. 25224420

[17] Guettiti H, Ennaifer E, Attia L, Chelly D, Alaya N, Aissa R, et al Pre-vaccination prevalence and genotype distribution of human papillomavirus infection among women from urban Tunis: a cross-sectional study. Asian Pacific Journal of Cancer Prevention. 2014;15(21):9361–9365. 25422225 10.7314/apjcp.2014.15.21.9361

[18] Hassen E1, Remadi S, Chouchane L. Detection and molecular typing of human papillomaviruses: prevalence of cervical infection in the Tunisian central region. Tunis Med. 1999 Oct;77(10):497–502. 10670281

[19] Bethesda System 2001. Acta Cytologica. 2001;45(6):1077–1078.

[20] World Health Organization (2009): Human papillomavirus laboratory manual. 1st eds Unger E. R, Dillner J, Zhou T. Geneva, Switzerland.

[21] Clifford G, Gallus S, Herrero R, Muñoz N, Snijders P, Vaccarella S, et al Worldwide distribution of human papillomavirus types in cytologically normal women in the International Agency for Research on Cancer HPV prevalence surveys: a pooled analysis. The Lancet. 2005;366(9490):991–998. 10.1016/S0140-6736(05)67069-9 16168781

[22] Bruni L, Diaz M, Castellsagué X, Ferrer E, Bosch F, de Sanjosé S. Cervical Human Papillomavirus Prevalence in 5 Continents: Meta‐Analysis of 1 Million Women with Normal Cytological Findings. The Journal of Infectious Diseases. 2010;202(12):1789–1799. 10.1086/657321 21067372

[23] Ogembo R, Gona P, Seymour A, Park H, Bain P, Maranda L, et al Prevalence of Human Papillomavirus Genotypes among African Women with Normal Cervical Cytology and Neoplasia: A Systematic Review and Meta-Analysis. PLOS ONE. 2015;10(4):e0122488 10.1371/journal.pone.0122488 25875167 PMC4396854

[24] El Mzibri M, Alhamany Z, Kharbach A, Malihy A, Abouqal R, Jaddi H, et al Prevalence of human papillomavirus genotype among Moroccan women during a local screening program. J Infect Dev Ctries. 2010;4(11). 10.3855/jidc.781 21252451

[25] Hammouda D, Clifford G, Pallardy S, Ayyach G, Chékiri A, Boudrich A, et al Human papillomavirus infection in a population-based sample of women in Algiers, Algeria. International Journal of Cancer. 2010;128(9):2224–2229. 10.1002/ijc.25539 20607828

[26] Shaltout M, Sallam H, AbouSeeda M, Moiety F, Hemeda H, Ibrahim A, et al Prevalence and type distribution of human papillomavirus among women older than 18 years in Egypt: a multicenter, observational study. International Journal of Infectious Diseases. 2014;29:226–231. 10.1016/j.ijid.2014.07.029 25447728

[27] Milutin Gašperov N, Sabol I, Matovina M, Spaventi Š, Grce M. Detection and Typing of Human Papillomaviruses Combining Different Methods: Polymerase Chain Reaction, Restriction Fragment Length Polymorphism, Line Probe Assay and Sequencing. Pathology & Oncology Research. 2008;14(4):355–363. 18752054 10.1007/s12253-008-9084-2

[28] Mbulawa Z, Coetzee D, Williamson A. Human papillomavirus prevalence in South African women and men according to age and human immunodeficiency virus status. BMC Infect Dis. 2015;15(1). 10.1186/s12879-015-1181-8 PMC4624185 26502723

[29] Chan P, Ho W, Wong M, Chang A, Chor J, Yu M. Epidemiologic risk profile of infection with different groups of human papillomaviruses. J Med Virol. 2009;81(9):1635–1644. 10.1002/jmv.21575 19623668

[30] Caiyan X, Weiyuan Z, Minghui W, Songwen Z. Prevalence and risk factors of lower genital tract infections among women in Beijing, China. Journal of Obstetrics and Gynaecology Research. 2011;38(1):310–315. 10.1111/j.1447-0756.2011.01624.x 21827575

[31] Brismar-Wendel S, Froberg M, Hjerpe A, Andersson S, Johansson B. Age-specific prevalence of HPV genotypes in cervical cytology samples with equivocal or low-grade lesions. Br J Cancer. 2009;101(3):511–517. 10.1038/sj.bjc.6605165 19623178 PMC2720239

[32] Grainge M, Seth R, Guo L, Neal K, Coupland C, Vryenhoef P, et al Cervical Human Papillomavirus Screening among Older Women. Emerg Infect Dis. 2005;11(11):1680–1685. 16318718 10.3201/eid1111.050575 PMC3367359

[33] Gonzalez P, Hildesheim A, Rodriguez A, Schiffman M, Porras C, Wacholder S, et al Behavioral/Lifestyle and Immunologic Factors Associated with HPV Infection among Women Older Than 45 Years. Cancer Epidemiology Biomarkers & Prevention. 2010;19(12):3044–3054. 10.1158/1055-9965.EPI-10-0645 PMC3703390 20952561

[34] Smith J, Melendy A, Rana R, Pimenta J. Age-Specific Prevalence of Infection with Human Papillomavirus in Females: A Global Review. Journal of Adolescent Health. 2008;43(4):S5.e1–S5.e62. 18809145 10.1016/j.jadohealth.2008.07.009

[35] Hildesheim A, Gravitt P, Schiffman M, Kurman R, Barnes W, Jones S, et al Determinants of Genital Human Papillomavirus Infection in Low-Income Women in Washington, D.C. Sexually Transmitted Diseases. 1993;20(5):279–285. 8235926 10.1097/00007435-199309000-00008

[36] Bauer H, Hildesheima A, Schiffman M, Glass A, Rush B, Scott D, et al Determinants of Genital Human Papillomavirus Infection in Low-Risk Women in Portland, Oregon. Sexually Transmitted Diseases. 1993;20(5):274–278. 8235925 10.1097/00007435-199309000-00007

[37] Mitchell S, Sekikubo M, Biryabarema C, Byamugisha J, Steinberg M, Jeronimo J, et al Factors associated with high-risk HPV positivity in a low-resource setting in sub-Saharan Africa. American Journal of Obstetrics and Gynecology. 2014;210(1):81.e1–81.e7. 23999419 10.1016/j.ajog.2013.08.038

[38] Moralejo D. Smoking increased risk of cervical cancer, independent of infection with high-risk HPV types. Evidence-Based Nursing. 2009;12(4):122–122. 10.1136/ebn.12.4.122 19779089

[39] Coker A, Bond S, Williams A, Gerasimova T, Pirisi L. Active and passive smoking, high-risk human papillomaviruses and cervical neoplasia. Cancer Detection and Prevention. 2002;26(2):121–128. 12102146 10.1016/s0361-090x(02)00039-9

[40] Liu Z, Liu W, Liu Y, Ye X, Chen S. Multiple Sexual Partners as a Potential Independent Risk Factor for Cervical Cancer: a Meta-analysis of Epidemiological Studies. Asian Pacific Journal of Cancer Prevention. 2015;16(9):3893–3900. 25987056 10.7314/apjcp.2015.16.9.3893

[41] Ojiyi E, Dike I, Okeudo C, Ejikem C, Nzewuihe A, Agbata A. Local risk factors in genital human papilloma Virus Infection in cervical smears. Annals of Medical and Health Sciences Research. 2013;3(4):529 10.4103/2141-9248.122082 24380003 PMC3868118

[42] Shi R, Devarakonda S, Liu L, Taylor H, Mills G. Factors associated with genital human papillomavirus infection among adult females in the United States, NHANES 2007–2010. BMC Research Notes. 2014;7(1):544. 25134828 10.1186/1756-0500-7-544 PMC4141114

