# Supplementary material for: Prevalence, Genotype Distribution and Risk Factors for Cervical Human Papillomavirus Infection in the Grand Tunis Region, Tunisia
Source: PLoS One. 2016 Jun 14;11(6):e0157432. doi: 10.1371/journal.pone.0157432 (PMC4907453; doi:10.1371/journal.pone.0157432)
Supplement: S1 Table — (DOC) [file pone.0157432.s001.doc]

**S1 Table. Comparison between Positive betaglobin and Negative betaglobin samples.**

|  | **Positive betaglobin** | **Negative betaglobin** | p |
| --- | --- | --- | --- |
| **Governorate** | | | |
| Ariana | 20,6% | 15,8% | 0.412 |
| Manouba | 14,5% | 22,8% |
| Ben Arous | 25,5% | 22,8% |
| Tunis | 39,4% | 38,6% |
| **Age** | | | |
| <=30 | 10,8% | 25,0% | 0.058 |
| [30-40[ | 35,5% | 32,1% |
| [40-50[ | 39,2% | 32,1% |
| >50 | 14,5% | 10,7% |
| **Education level** | | | |
| Illiterate | 17,2% | 12,3% | 0.624 |
| Primary level | 50,5% | 52,6% |
| Secondary and high level | 32,3% | 35,1% |
| **Marital status** | | | |
| Married | 94,8% | 93,0% | 0.385 |
| Widowed, divorced, separated, never married | 5,2% | 7,0% |
| **Income Index** | | | |
| Below Poverty | 56.2% | 50.9% | 0.195 |
| Above Poverty Intermediate Index | 37.2% | 35.1% |
| Above Poverty High Index | 6.6% | 14% |
| **Housing Type** | | | |
| Good | 24,8% | 29,8% | 0.257 |
| Bad | 75,2% | 70,2% |
| **Occupation** | | | |
| unemployed | 35,7% | 28,6% | 0.376 |
| With regular job | 64,3% | 71,4% |
| **Tobacco use** | | | |
| Yes | 11,4% | 10,5% | 0.529 |
| No | 88,6% | 89,5% |
| Menopause | | | |
| Yes | 15.7 | 10.5 | 0.213 |
| No | 84.3% | 85.9% |
| Pregnancy | | | |
| Yes | 2.9% | 7.5% | 0.281 |
| No | 96.7% | 9.2% |
| Contraception | | | |
| Yes | 72.6% | 77.2% | 0.291 |
| No | 27.4% | 22.8% |
| Medical history of chronic disease | | | |
| Yes | 31.4% | 31.6% | 0.454 |
| No | 68.6% | 68.4% |
| Surgical history | | | |
| Yes | 38.9% | 43.9% | 0.286 |
| No | 61.1% | 56.1% |
| casual sexual relation the last 12 months | | | |
| Yes | 11.4% | 7% | 0.969 |
| No | 88.6% | 93% |
| Sexually transmitted infection history | | | |
| Yes | 26.8% | 24.6% | 0.329 |
| No | 73.2% | 75.4% |
| Multiple sexual intercourse of partner | | | |
| Yes | 14.8% | 14% | 0.835 |
| No | 85.2% | 86% |
| age at first sexual intercourse | | | |
| ≤ 18 years | 12% | 14% | 0.401 |
| >18 years | 88% | 86% |
| Multiple sexual partners | | | |
| Yes | 27.4% | 21.1% | 0.271 |
| No | 72.6% | 78.9% |
